# Casirivimab and Imdevimab Treatment Reduces Viral Load and Improves Clinical Outcomes in Seropositive Hospitalized COVID-19 Patients with Nonneutralizing or Borderline Neutralizing Antibodies

**DOI:** 10.1128/mbio.01699-22

**Published:** 2022-10-18

**Authors:** Andrea T. Hooper, Selin Somersan-Karakaya, Shane E. McCarthy, Eleftherios Mylonakis, Shazia Ali, Jingning Mei, Rafia Bhore, Adnan Mahmood, Gregory P. Geba, Paula Dakin, David M. Weinreich, George D. Yancopoulos, Gary A. Herman, Jennifer D. Hamilton

**Affiliations:** a Regeneron Pharmaceuticals, Inc., Tarrytown, New York, USA; b Brown University, Providence, Rhode Island, USA; McMaster University

**Keywords:** COVID-19, anti-SARS-CoV-2 serostatus, hospitalized, monoclonal antibodies, casirivimab and imdevimab, neutralizing antibodies

## Abstract

We conducted a *post hoc* analysis in seropositive patients who were negative or borderline for functional neutralizing antibodies (NAbs) against severe acute respiratory syndrome coronavirus 2 (SARS-CoV-2) at baseline from a phase 1, 2, and 3 trial of casirivimab and imdevimab (CAS+IMD) treatment in hospitalized coronavirus disease 2019 (COVID-19) patients on low-flow or no supplemental oxygen prior to the emergence of Omicron-lineage variants. Patients were randomized to a single dose of 2.4 g CAS+IMD, 8.0 g CAS+IMD, or placebo. Patients seropositive for anti-SARS-CoV-2 antibodies at baseline were analyzed by their baseline neutralizing antibody status. At baseline, 20.6% (178/864) of seropositive patients were negative or borderline for neutralizing antibodies, indicating negative or very low functionally neutralizing anti-SARS-CoV-2 antibodies. CAS+IMD reduced viral load in patients who were negative or borderline for neutralizing antibodies versus placebo, but not in patients who were positive for neutralizing antibodies. In patients who were negative or borderline for neutralizing antibodies, we observed a trend in reduction of the proportion of patients who died or required mechanical ventilation, as well as in all-cause mortality, by day 29 with CAS+IMD versus placebo. The proportions of patients who died or required mechanical ventilation from days 1 to 29 were 19.1% in the placebo group and 10.9% in the CAS+IMD combined-dose group, and the proportions of patients who died (all-cause mortality) from days 1 to 29 were 16.2% in the placebo group and 9.1% in the CAS+IMD combined-dose group. In patients who were positive for neutralizing antibodies, no measurable harm or benefit was observed in either the proportion of patients who died or required mechanical ventilation or the proportion of patients who died (all-cause mortality). In hospitalized COVID-19 patients on low-flow or no supplemental oxygen, CAS+IMD reduced viral load, the risk of death or mechanical ventilation, and all-cause mortality in seropositive patients who were negative or borderline for neutralizing antibodies.

## INTRODUCTION

Severe acute respiratory syndrome coronavirus 2 (SARS-CoV-2) continues to evolve, and data have shown that complete coronavirus disease 2019 (COVID-19) vaccination with a booster is protective against symptomatic disease and severe COVID-19 (1–3). As the prevalence of SARS-CoV-2-seropositive individuals rises due to both vaccination and previous infection (4), it is important to understand whether there is a subset of patients with COVID-19 who have antibodies against SARS-CoV-2 who could benefit from anti-SARS-CoV-2 monoclonal antibody treatment.

Monoclonal antibody therapeutics benefit patients across the spectrum of COVID-19 disease severity (5), as well as individuals who can neither receive nor respond to vaccines (6, 7). Casirivimab and imdevimab (CAS+IMD) is a combination of two neutralizing monoclonal antibodies that bind nonoverlapping epitopes of the SARS-CoV-2 spike protein receptor-binding domain (8). Prior to the emergence of the Omicron variant, CAS+IMD was shown to be effective in the treatment of outpatients with COVID-19 (9) by reducing viral load, decreasing risk of hospitalization or death, and decreasing symptom duration, as well as demonstrating efficacy in the prevention of COVID-19 (10).

The efficacy and safety of CAS+IMD in hospitalized patients with COVID-19 were demonstrated in an open-label platform trial in the United Kingdom (RECOVERY) (11) as well as a phase 1, 2, and 3 double-blinded, placebo-controlled trial in patients on low-flow or no supplemental oxygen (Study 2066; ClinicalTrials registration no. NCT04426695) (12). In the RECOVERY study, CAS+IMD reduced mortality in hospitalized patients with COVID-19, but this benefit was observed only in patients who were seronegative (i.e., had no measurable antibody immunity to SARS-CoV-2) at baseline (11). Consistent with these data, clinical benefit, including less likelihood of death or need for mechanical ventilation, lower all-cause mortality, and improved rates of hospital discharge, were observed in the overall population in study 2066, driven by the benefit in seronegative patients and with no benefit or harm observed in seropositive patients (12).

In the current landscape, where the majority of the population is vaccinated against COVID-19 and/or has a history of SARS-CoV-2 infection, the estimated anti-SARS-CoV-2 seropositivity rate in the United States is 94.7% (13). Thus, it is important to better understand the potential role of CAS+IMD treatment in seropositive patients. Although seropositive patients have detectable anti-SARS-CoV-2 antibodies, it was hypothesized that the neutralizing function of these antibodies in a subset of hospitalized patients may be impaired. We questioned whether treatment with CAS+IMD may also provide clinical benefit in certain subsets of seropositive hospitalized patients with COVID-19. To further investigate this question, we conducted a *post hoc* analysis of seropositive patients from study 2066 who were negative or borderline at baseline for neutralizing antibodies (NAbs) against SARS-CoV-2 compared with those patients with measurable baseline neutralizing activity. While CAS+IMD has markedly diminished neutralization against the Omicron variant (14) and is not currently authorized in any geographic regions where infection is likely to have been caused by a nonsusceptible SARS-CoV-2 variant (15), study 2066 was conducted prior to the emergence of Omicron and subsequent variants and therefore allowed us to evaluate the benefit of CAS+IMD in a subset of hospitalized patients with susceptible strains of SARS-CoV-2.

## RESULTS

### Patient characteristics by neutralizing antibody status.

As of 9 April 2021, prior to the emergence of Delta- or Omicron-lineage variants, 2,053 patients from phases 1, 2, and 3 on low-flow or no supplemental oxygen were randomized into the study, of whom 2,007 were treated and included in the full analysis set (FAS) (see Fig. S1 in the supplemental material). Of those, 1,759 tested positive for SARS-CoV-2 by central lab quantitative reverse transcription-PCR (RT-qPCR) testing, constituting the modified full analysis set (mFAS). Of these patients, a total of 864 (49.1%) were seropositive at baseline (seropositive mFAS) comprising the primary population for the presented analysis, including 560 patients in the CAS+IMD combined-dose group and 304 patients in the placebo group. Anti-SARS-CoV-2 seropositivity was defined using a composite of three individual assays, as detailed in Materials and Methods.

10.1128/mbio.01699-22.7 FIG S1 Flow diagram for the phase 1, 2, and 3 populations receiving low-flow or no supplemental oxygen. Download FIG S1, PDF file, 0.1 MB. Copyright © 2022 Hooper et al. 2022 Hooper et al. https://creativecommons.org/licenses/by/4.0/ This content is distributed under the terms of the Creative Commons Attribution 4.0 International license.

Although antibodies against SARS-CoV-2 are detected in seropositive individuals in this study, the neutralizing function of those antibodies is not characterized by the three serology assays used to determine serostatus. Therefore, neutralizing antibody status was determined using a high-throughput clinical test that measures the capacity of patient serum samples to neutralize recombinant vesicular stomatitis virus (VSV) encoding the SARS-CoV-2 spike glycoprotein (16). Using this assay, seropositive patients were characterized as positive, negative, or borderline for SARS-CoV-2 functional neutralizing antibodies. At baseline, 20.6% (178/864) of seropositive patients (seropositive mFAS) were negative or borderline for neutralizing antibodies, including 110 patients in the CAS+IMD combined-dose group and 68 patients in the placebo group. Further serological characterization of seropositive patients by neutralizing antibody status is presented in Tables S1 and S2.

10.1128/mbio.01699-22.1 TABLE S1 Characterization of neutralization status in seropositive patients by individual serology assays. Download Table S1, file, 0.1 MB. Copyright © 2022 Hooper et al. 2022 Hooper et al. https://creativecommons.org/licenses/by/4.0/ This content is distributed under the terms of the Creative Commons Attribution 4.0 International license.

10.1128/mbio.01699-22.2 TABLE S2 Characterization of subgroup seropositivity by neutralization status. Download Table S2, PDF file, 0.1 MB. Copyright © 2022 Hooper et al. 2022 Hooper et al. https://creativecommons.org/licenses/by/4.0/ This content is distributed under the terms of the Creative Commons Attribution 4.0 International license.

**FIG 1:**
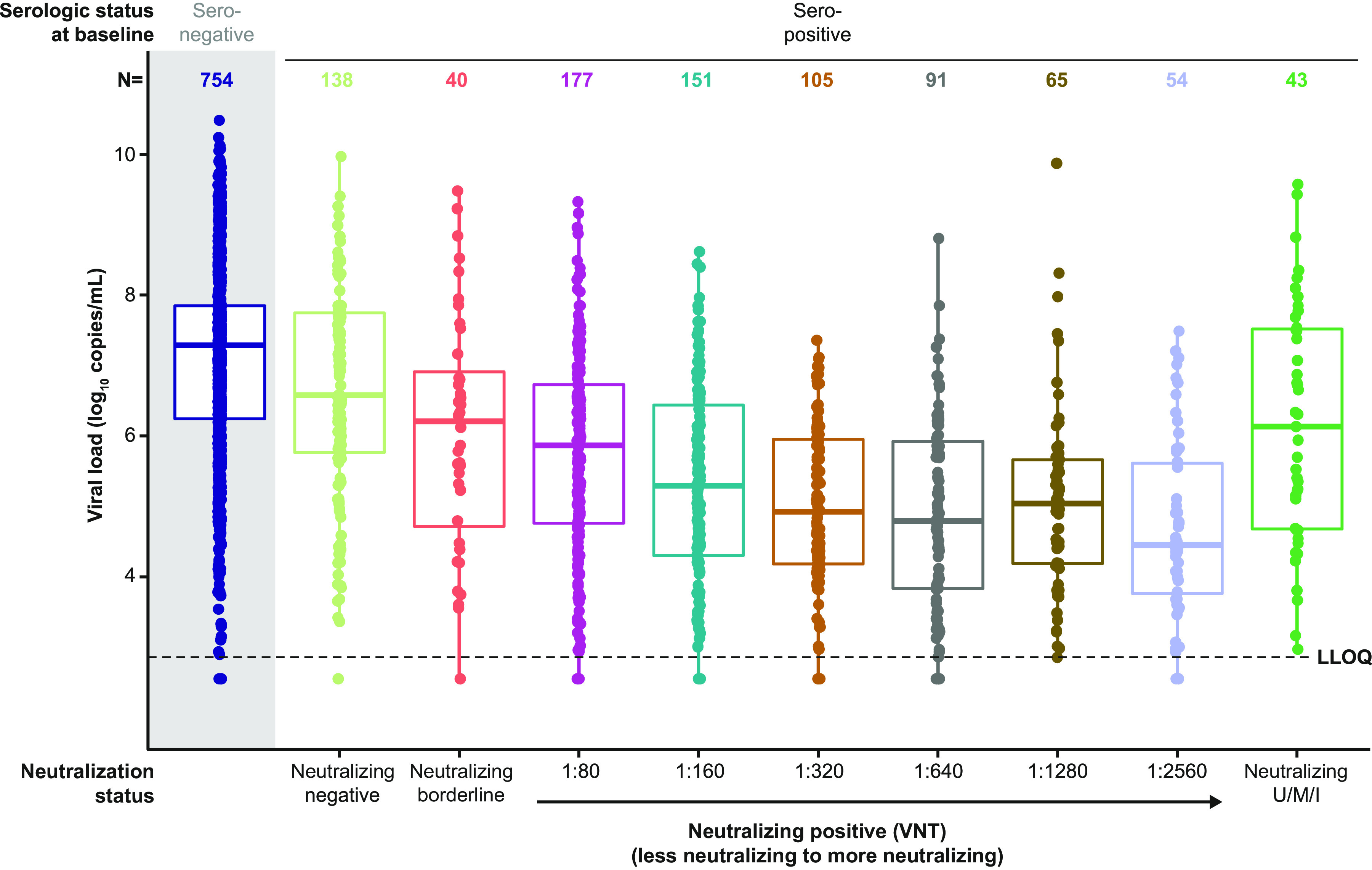
Viral load by baseline neutralizing antibody titer. Serostatus was determined using composite serostatus based on Euroimmun anti-spike S1 IgA and IgG assays and the Abbott anti-nucleocapsid IgG assay. Seropositive is defined as positive in any test; seronegative is defined as negative in all available tests. Neutralizing titer was determined by IMMUNO-COV neutralization assay in seropositive patients only; the seronegative group was not tested in the neutralizing assay. Dots represent individual patient data; boxes represent median and interquartile range. The LLOQ for the viral load determination RT-qPCR assay was 2.85-log_10_ copies/mL. mFAS presented. Ig, immunoglobulin; LLOQ, lower limit of quantification; mFAS, modified full analysis set; RT-qPCR, quantitative reverse transcription-PCR; VNT, viral neutralizing titer; U, M, and I, unknown, missing, and indeterminant, respectively.

Baseline demographics and characteristics differed slightly among those who were negative or borderline for neutralizing antibodies from those who were positive for neutralizing antibodies. Patients who were negative or borderline for neutralizing antibodies were older (64.5 versus 60.0 years), had higher baseline viral loads (median values of 6.5- versus 5.2-log_10_ copies/mL), included fewer Hispanic or Latino patients (24.2% versus 39.8%), and had a greater proportion of immunocompromised patients (24.7% versus 12.9%) than patients who were positive for neutralizing antibodies, respectively (Table 1). Furthermore, seropositive patients with lower neutralizing titers at baseline exhibited higher baseline viral loads (Fig. 1).

**TABLE 1:** Demographics and baseline characteristics in seropositive patients by baseline neutralizing antibody status^*a*^^,^^*c*^

Characteristic	Data for patients who received:				
	Placebo	CAS+IMD (2.4 g i.v.)	CAS+IMD (8.0 g i.v.)	CAS+IMD (combined doses)	Total
Negative or borderline baseline neutralizing antibody status					
No. of patients	68	57	53	110	178
Age (median [range] [yrs])	66.0 (24–100)	61.0 (35–94)	63.0 (37–95)	62.5 (35–95)	64.5 (24–100)
No. (%) of patients ≥65 yrs	40 (58.8)	26 (45.6)	23 (43.4)	49 (44.5)	89 (50.0)
Male sex (no. [%])	38 (55.9)	31 (54.4)	28 (52.8)	59 (53.6)	97 (54.5)
Race (no. [%])					
White	50 (73.5)	35 (61.4)	43 (81.1)	78 (70.9)	128 (71.9)
Black or African American	8 (11.8)	8 (14.0)	4 (7.5)	12 (10.9)	20 (11.2)
Asian	2 (2.9)	3 (5.3)	1 (1.9)	4 (3.6)	6 (3.4)
American Indian or Alaska Native	0	1 (1.8)	0	1 (0.9)	1 (0.6)
Native Hawaiian or Pacific Islander	0	1 (1.8)	0	1 (0.9)	1 (0.6)
Unknown	2 (2.9)	5 (8.8)	2 (3.8)	7 (6.4)	9 (5.1)
Not reported	6 (8.8)	4 (7.0)	3 (5.7)	7 (6.4)	13 (7.3)
Ethnicity (no. [%])					
Hispanic or Latino	18 (26.5)	18 (31.6)	7 (13.2)	25 (22.7)	43 (24.2)
Not Hispanic or Latino	47 (69.1)	39 (68.4)	43 (81.1)	82 (74.5)	129 (72.5)
Not reported	3 (4.4)	0	3 (5.7)	3 (2.7)	6 (3.4)
Wt (mean [SD] [kg])	89.40 (22.794)	85.14 (22.412)	91.71 (27.033)	88.30 (24.852)	88.72 (24.027)
Body mass index (kg/m^2^)					
Mean (SD)	30.94 (8.034)	29.79 (6.684)	31.20 (8.840)	30.45 (7.772)	30.64 (7.851)
No. (%) ≥30	33 (48.5)	22 (38.6)	27 (50.9)	49 (44.5)	82 (46.1)
No. of days of COVID-19 illness prior to baseline (median [Q1, Q3])	5.0 (4.0, 8.0)	7.0 (4.0, 8.0)	6.0 (4.0, 7.0)	6.0 (4.0, 8.0)	6.0 (4.0, 8.0)
Baseline viral load					
Median (Q1, Q3 [log_10_ copies/mL])	6.6 (5.1, 7.7)	6.5 (5.6, 7.5)	6.6 (5.6, 7.7)	6.5 (5.6, 7.7)	6.5 (5.6, 7.7)
No. (%) with >10^4^ copies/mL	62 (91.2)	53 (93.0)	48 (90.6)	101 (91.8)	163 (91.6)
No. (%) with >10^6^ copies/mL	42 (61.8)	38 (66.7)	37 (69.8)	75 (68.2)	117 (65.7)
Concomitant medications (no. [%])					
Remdesivir	40 (58.8)	33 (57.9)	39 (73.6)	72 (65.5)	112 (62.9)
Systemic corticosteroids	50 (73.5)	44 (77.2)	47 (88.7)	91 (82.7)	141 (79.2)
Use of supplemental oxygen (no. [%])	53 (77.9)	40 (70.2)	34 (64.2)	74 (67.3)	127 (71.3)
Immunocompromised (no. [%])^*b*^	20 (29.4)	11 (19.3)	13 (24.5)	24 (21.8)	44 (24.7)
Positive baseline neutralizing antibody status					
No. of patients	222	213	208	421	643
Age (median [range] [yrs])	60.0 (22–95)	59.0 (20–90)	60.5 (20–90)	60.0 (20–90)	60.0 (20–95)
No. (%) of patients ≥65 yrs	82 (36.9)	70 (32.9)	84 (40.4)	154 (36.6)	236 (36.7)
Male sex (no. [%])	123 (55.4)	121 (56.8)	119 (57.2)	240 (57.0)	363 (56.5)
Race (no. [%])					
White	132 (59.5)	136 (63.8)	123 (59.1)	259 (61.5)	391 (60.8)
Black or African American	30 (13.5)	24 (11.3)	29 (13.9)	53 (12.6)	83 (12.9)
Asian	9 (4.1)	12 (5.6)	12 (5.8)	24 (5.7)	33 (5.1)
American Indian or Alaska Native	8 (3.6)	5 (2.3)	12 (5.8)	17 (4.0)	25 (3.9)
Native Hawaiian or Pacific Islander	2 (0.9)	0	2 (1.0)	2 (0.5)	4 (0.6)
Unknown	15 (6.8)	13 (6.1)	13 (6.3)	26 (6.2)	41 (6.4)
Not reported	26 (11.7)	23 (10.8)	17 (8.2)	40 (9.5)	66 (10.3)
Ethnicity (no. [%])					
Hispanic or Latino	90 (40.5)	86 (40.4)	80 (38.5)	166 (39.4)	256 (39.8)
Not Hispanic or Latino	123 (55.4)	117 (54.9)	121 (58.2)	238 (56.5)	361 (56.1)
Not reported	9 (4.1)	10 (4.7)	7 (3.4)	17 (4.0)	26 (4.0)
Wt (mean [SD] [kg])	89.13 (24.328)	89.78 (24.988)	90.33 (25.582)	90.05 (25.256)	89.73 (24.924)
Body mass index (kg/m^2^)					
Mean (SD)	31.62 (7.510)	31.91 (8.112)	31.72 (8.189)	31.82 (8.141)	31.75 (7.921)
No. (%) ≥30	118 (53.2)	106 (49.8)	106 (51.0)	212 (50.4)	330 (51.3)
No. of days of COVID-19 illness prior to baseline (median [Q1, Q3])	7.0 (5.0, 8.0)	7.0 (5.0, 8.0)	7.0 (5.0, 8.0)	7.0 (5.0, 8.0)	7.0 (5.0, 8.0)
Baseline viral load					
Median (Q1, Q3 [log_10_ copies/mL])	5.1 (4.3, 6.1)	5.1 (4.2, 6.2)	5.4 (4.2, 6.4)	5.2 (4.2, 6.3)	5.2 (4.2, 6.2)
No. (%) with >10^4^ copies/mL	184 (82.9)	171 (80.3)	164 (78.8)	335 (79.6)	519 (80.7)
No. (%) with >10^6^ copies/mL	64 (28.8)	62 (29.1)	67 (32.2)	129 (30.6)	193 (30.0)
Concomitant medications (no. [%])					
Remdesivir	139 (62.6)	120 (56.3)	125 (60.1)	245 (58.2)	384 (59.7)
Systemic corticosteroids	177 (79.7)	166 (77.9)	181 (87.0)	347 (82.4)	524 (81.5)
Use of supplemental oxygen (no. [%])	177 (79.7)	165 (77.5)	175 (84.1)	340 (80.8)	517 (80.4)
Immunocompromised (no. [%])^*b*^	30 (13.5)	32 (15.0)	21 (10.1)	53 (12.6)	83 (12.9)

a Seropositive mFAS presented.

b Immunocompromised patients include those with immunological diseases, are immunosuppressed, or have immunodeficiencies.

c CAS+IMD, casirivimab and imdevimab; COVID-19, coronavirus disease 2019; i.v., intravenous; mFAS, modified full analysis set; Q, quartile.

### Virological efficacy.

**FIG 2:**
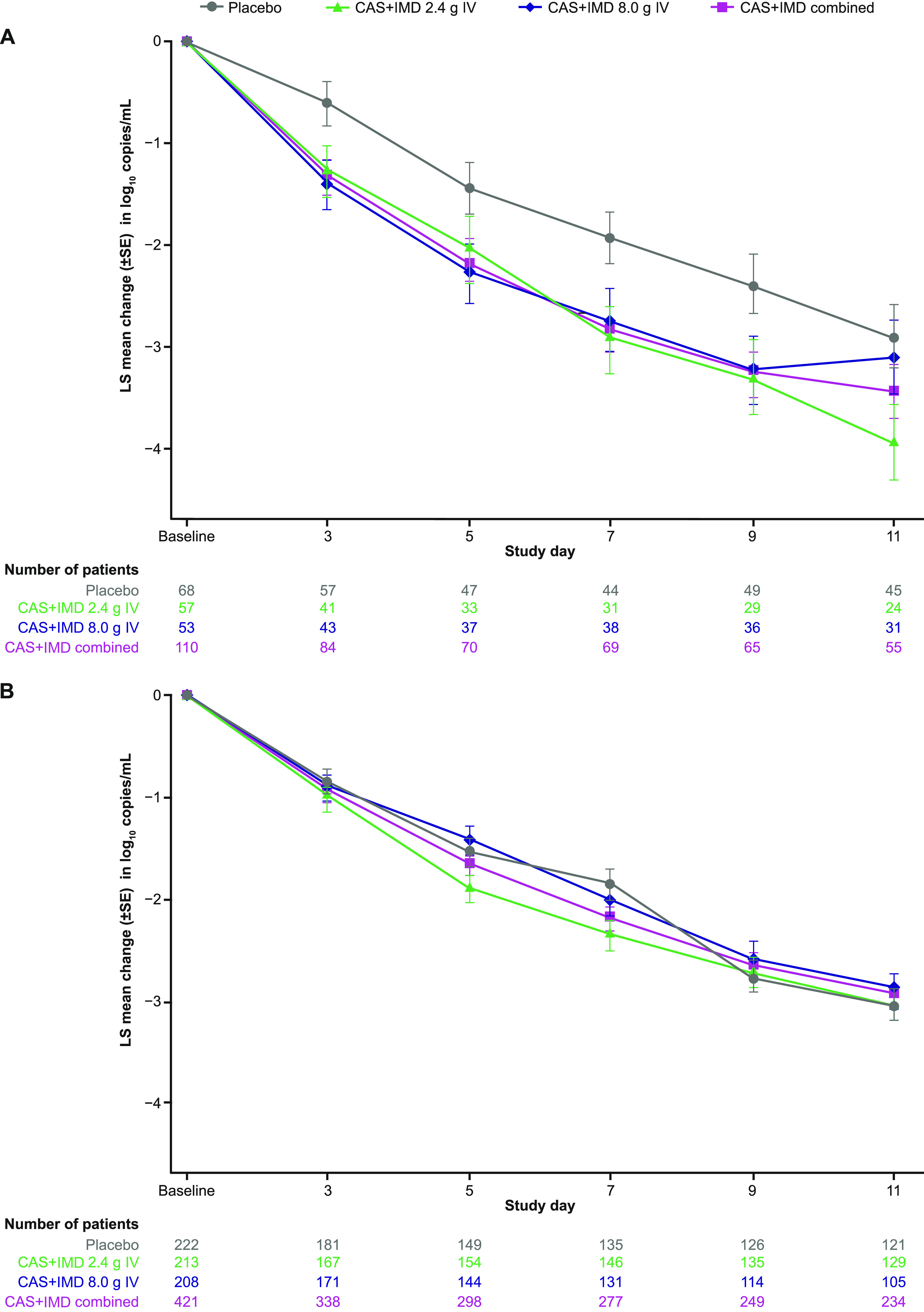
Change from baseline in viral load in seropositive patients by baseline neutralizing antibody status. (A) LS mean viral load following administration of CAS+IMD (2.4 g, 8.0 g, or combined analysis of 2.4 g and 8.0 g) or placebo for seropositive patients who were negative or borderline for neutralizing antibodies. (B) Same as panel A but for seropositive patients who were positive for neutralizing antibodies. Seropositive mFAS presented. CAS+IMD, casirivimab and imdevimab; IV, intravenous; mFAS, modified full analysis set; LS, least squares.

In seropositive patients on low-flow or no supplemental oxygen, treatment with CAS+IMD reduced viral load, relative to placebo, at all time points evaluated in patients who were negative or borderline for neutralizing antibodies (Fig. 2A) but not in patients with measurable neutralizing activity (Fig. 2B). In patients who were negative or borderline for neutralizing antibodies, a significant reduction in viral load with CAS+IMD versus placebo was observed as early as the first follow-up time point on day 3 and continued through day 11 (Table S3). Least-squares (LS) mean time-weighted average (TWA) daily change in viral load from baseline (day 1) through day 3 was −0.27-log_10_ copies/mL (95% confidence interval [CI], −0.48 to −0.05) in the placebo group compared with −0.66-log_10_ copies/mL (95% CI, −0.83 to −0.48) in the CAS+IMD combined-dose group, with an LS mean difference versus placebo of −0.39- log_10_ copies/mL (95% CI, −0.66 to −0.11; nominal *P = *0.0061). LS mean TWA daily change in viral load from baseline through day 11 was −1.33-log_10_ copies/mL (95% CI, −1.64 to −1.03) in the placebo group compared with −1.87-log_10_ copies/mL (95% CI, −2.11 to −1.63) in the CAS+IMD combined-dose group, with an LS mean difference versus placebo of −0.54-log_10_ copies/mL (95% CI, −0.93 to −0.15; nominal *P = *0.0067).

10.1128/mbio.01699-22.3 TABLE S3 Time-weighted average change in viral load from baseline in seropositive patients by baseline neutralizing antibody status. Download Table S3, PDF file, 0.1 MB. Copyright © 2022 Hooper et al. 2022 Hooper et al. https://creativecommons.org/licenses/by/4.0/ This content is distributed under the terms of the Creative Commons Attribution 4.0 International license.

### Clinical efficacy: death or mechanical ventilation.

**FIG 3:**
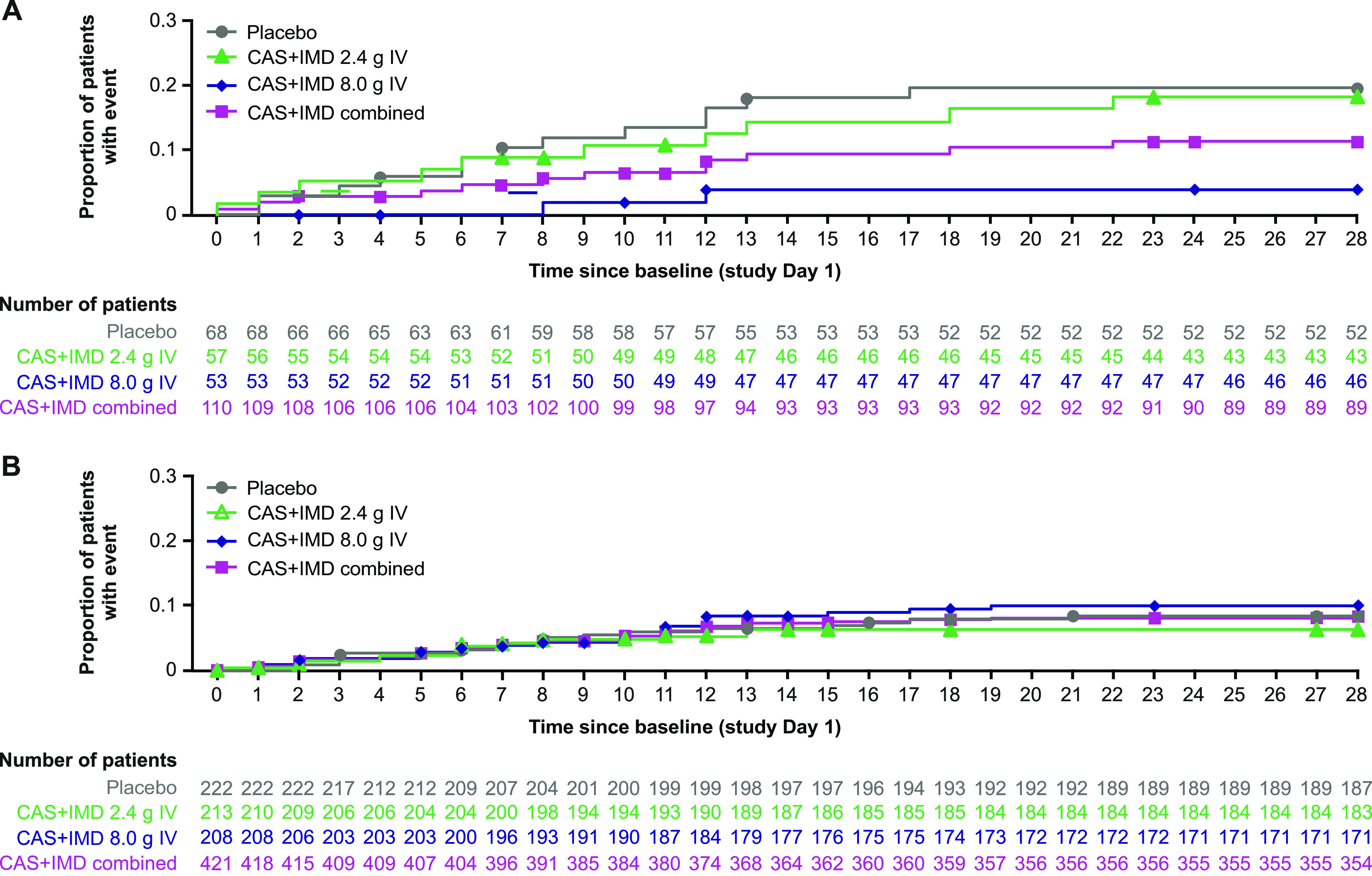
Cumulative incidence of death or mechanical ventilation in seropositive patients by baseline neutralizing antibody status. Kaplan-Meier curves for the proportion of patients who died or required mechanical ventilation through study day 29 after administration of CAS+IMD (2.4 g, 8.0 g, or combined analysis of 2.4 g and 8.0 g) or placebo in patients who were negative or borderline for neutralizing antibodies (A) or patients who were positive for neutralizing antibodies (B). Symbols indicate censoring. Seropositive mFAS presented. CAS+IMD, casirivimab and imdevimab; IV, intravenous; mFAS, modified full analysis set.

Though it was limited by small numbers, in seropositive patients on low-flow or no supplemental oxygen, a trend toward benefit in the proportion of patients who died or required mechanical ventilation was observed with CAS+IMD treatment versus placebo in patients who were negative or borderline for neutralizing antibodies (Fig. 3A). In this subset of patients, the proportion who died or required mechanical ventilation from days 1 to 29 was 19.1% (13/68) in the placebo group compared with 10.9% (12/110) in the CAS+IMD combined-dose group (relative risk reduction, 49.2%; nominal *P = *0.1125; Table S4).

10.1128/mbio.01699-22.4 TABLE S4 Clinical outcomes in seropositive patients by baseline neutralizing antibody status. Download Table S4, PDF file, 0.1 MB. Copyright © 2022 Hooper et al. 2022 Hooper et al. https://creativecommons.org/licenses/by/4.0/ This content is distributed under the terms of the Creative Commons Attribution 4.0 International license.

No measurable benefit or harm in the proportion of patients who died or required mechanical ventilation was observed in seropositive patients who were positive for neutralizing antibodies (Fig. 3B).

### Clinical efficacy: all-cause mortality.

**FIG 4:**
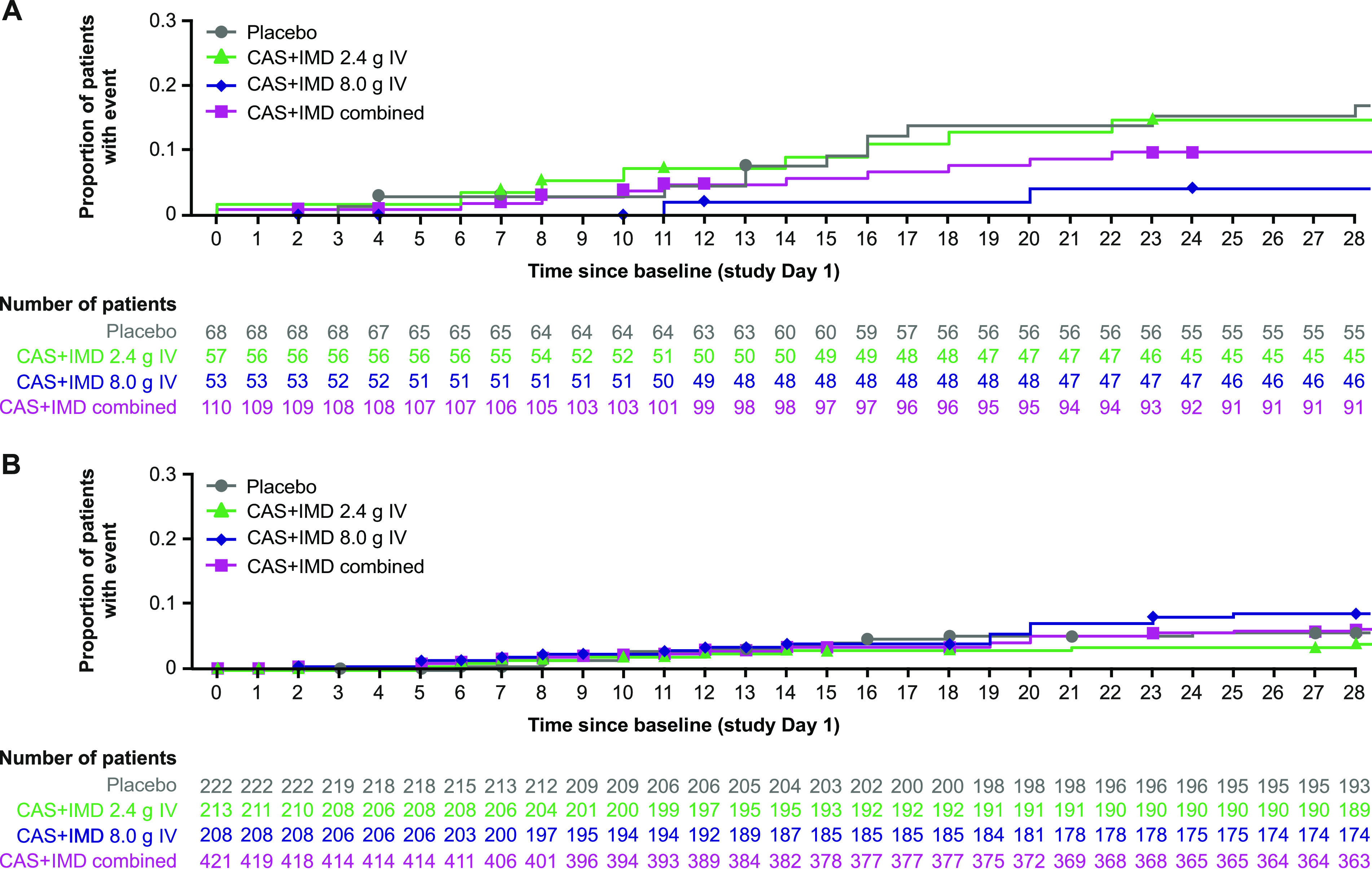
Cumulative incidence of death in seropositive patients by baseline neutralizing antibody status. Kaplan-Meier curves for the proportion of patients who died through study day 29 after administration of CAS+IMD (2.4 g, 8.0 g, or combined analysis of 2.4 g and 8.0 g) or placebo in patients who were negative or borderline for neutralizing antibodies (A) or patients who were positive for neutralizing antibodies (B). Symbols indicate censoring. Seropositive mFAS presented. CAS+IMD, casirivimab and imdevimab; IV, intravenous; mFAS, modified full analysis set.

Though it was also limited by small numbers, in seropositive patients on low-flow or no supplemental oxygen, a trend toward benefit in all-cause mortality was observed with CAS+IMD treatment versus placebo in patients who were negative or borderline for neutralizing antibodies (Fig. 4A). The proportion of these patients who died from days 1 to 29 was 16.2% (11/68) in the placebo group compared with 9.1% (10/110) in the CAS+IMD combined-dose group (relative risk reduction, 43.8%; nominal *P = *0.1190; Table S4).

No measurable benefit or harm in all-cause mortality was observed in seropositive patients who were positive for neutralizing antibodies (Fig. 4B).

### Safety.

While event rates were small, in the subset of patients who were negative or borderline for neutralizing antibodies, serious adverse events (SAEs) were reported by 30.9% (21/68) and 29.1% (32/110) in the placebo and CAS+IMD combined-dose groups, respectively (Table 2). In the subset of patients who were positive for neutralizing antibodies, SAEs were reported by 16.2% (36/222) and 14.7% (62/421) in the placebo and CAS+IMD combined-dose groups, respectively.

**TABLE 2:** Overview of TEAEs in seropositive patients by baseline neutralizing antibody status^*a*^^,^^*f*^*^,^*^*g*^

Patient status	Data for patients who received:			
	Placebo	CAS+IMD (2.4 g i.v.)	CAS+IMD (8.0 g i.v.)	CAS+IMD (combined doses)
Patients with negative or borderline baseline neutralizing antibodies				
No. of patients	68	57	53	110
Any TEAE^*b*^	21 (30.9)	20 (35.1)	16 (30.2)	36 (32.7)
Any grade 3 or 4 TEAE	14 (20.6)	14 (24.6)	12 (22.6)	26 (23.6)
Any treatment-emergent SAE	21 (30.9)	20 (35.1)	12 (22.6)	32 (29.1)
Any treatment-emergent AESI	0	2 (3.5)	5 (9.4)	7 (6.4)
Any treatment-emergent serious AESI	0	2 (3.5)	1 (1.9)	3 (2.7)
Any treatment-emergent AESI of infusion-related reactions (grade ≥2) through day 4^*c*^	0	2 (3.5)	4 (7.5)	6 (5.5)
Any treatment-emergent AESI of hypersensitivity reactions (grade ≥2) through day 29	0	0	2 (3.8)	2 (1.8)
Any TEAE leading to study infusion interruption^*d*^	0	0	0	0
Any TEAE leading to study infusion discontinuation^*e*^	0	0	0	0
Patients with positive baseline neutralizing antibody status				
No. of patients	222	213	208	421
Any TEAE^*b*^	38 (17.1)	34 (16.0)	37 (17.8)	71 (16.9)
Any grade 3 or 4 TEAE	28 (12.6)	22 (10.3)	23 (11.1)	45 (10.7)
Any treatment-emergent SAE	36 (16.2)	29 (13.6)	33 (15.9)	62 (14.7)
Any treatment-emergent AESI	1 (0.5)	4 (1.9)	3 (1.4)	7 (1.7)
Any treatment-emergent serious AESI	0	0	0	0
Any treatment-emergent AESI of infusion-related reactions (grade ≥ 2) through day 4^*c*^	1 (0.5)	3 (1.4)	2 (1.0)	5 (1.2)
Any treatment-emergent AESI of hypersensitivity reactions (grade ≥ 2) through day 29	0	1 (0.5)	1 (0.5)	2 (0.5)
Any TEAE leading to study infusion interruption^*d*^	0	1 (0.5)	1 (0.5)	2 (0.5)
Any TEAE leading to study infusion discontinuation^*e*^	0	0	0	0

a Seropositive mFAS presented.

b TEAEs collected include treatment-emergent SAEs, AESIs, and grade 3 and 4 TEAEs, as well as a*d hoc* or voluntarily reported TEAEs by some sites.

c Deemed treatment related as per investigator assessment.

d Infusion interruption. The administration of the infusion was interrupted before being completed but subsequently was restarted, and the full planned dose was administered.

e Infusion discontinuation. The administration of the infusion was stopped before being completed, and the full planned dose was not administered.

f AESI, adverse event of special interest; CAS+IMD, casirivimab and imdevimab; i.v., intravenous; SAE, serious adverse event; TEAE, treatment-emergent adverse event.

g All data represent number and percent unless otherwise specified.

In the subset of patients who were negative or borderline for neutralizing antibodies, more patients experienced adverse events that resulted in death in the placebo group than those in the CAS+IMD combined-dose group (17.6% [12/68] placebo versus 10.0% [11/110] CAS+IMD; Table S5), consistent with the treatment benefit highlighted in the efficacy section. Interestingly, this trend was not observed in the subset of patients who were positive for neutralizing antibodies (6.3% [14/222] placebo versus 7.1% [30/421] CAS+IMD).

10.1128/mbio.01699-22.5 TABLE S5 Adverse events leading to death in seropositive patients by baseline neutralizing antibody status. Download Table S5, PDF file, 0.1 MB. Copyright © 2022 Hooper et al. 2022 Hooper et al. https://creativecommons.org/licenses/by/4.0/ This content is distributed under the terms of the Creative Commons Attribution 4.0 International license.

In the subset of patients who were negative or borderline for neutralizing antibodies, no adverse events of special interest (AESIs; grade ≥ 2 infusion-related reactions and grade ≥ 2 hypersensitivity reactions) were reported in the placebo group, while AESIs were reported by 6.4% (7/110) of patients in the CAS+IMD combined-dose group (Table S6). In the subset of patients who were positive for neutralizing antibodies, AESIs were reported by 0.5% (1/222) and 1.7% (7/421) in the placebo and CAS+IMD combined-dose groups, respectively.

10.1128/mbio.01699-22.6 TABLE S6 Adverse events of special interest in seropositive patients by baseline neutralizing antibody status. Download Table S6, PDF file, 0.1 MB. Copyright © 2022 Hooper et al. 2022 Hooper et al. https://creativecommons.org/licenses/by/4.0/ This content is distributed under the terms of the Creative Commons Attribution 4.0 International license.

## DISCUSSION

The clinical benefit of CAS+IMD in hospitalized seronegative patients with COVID-19 was previously demonstrated in the RECOVERY study and the primary analysis of study 2066 (11, 12). In these studies, no clinical benefit was observed in patients with baseline seropositivity to SARS-CoV-2 with active treatment relative to placebo. Presumably, the endogenous neutralizing activity in patients mimics the activity of CAS+IMD; thus, viral measures and clinical outcomes in seropositive patients treated with placebo are similar to those treated with CAS+IMD. We now extend the findings from study 2066 by demonstrating that there is a subset of hospitalized patients who have detectable antibodies against SARS-CoV-2 (i.e., seropositive) but who may still benefit from treatment with anti-SARS-CoV-2 monoclonal antibody therapies, as their endogenous antibodies may not appropriately neutralize SARS-CoV-2 (i.e., those who were negative or borderline for neutralizing antibodies in an assay that measures the capability of patient serum to neutralize recombinant VSV encoding the SARS-CoV-2 spike glycoprotein).

In this *post hoc* analysis of hospitalized patients with COVID-19 on low-flow or no supplemental oxygen, we found that approximately 20% of seropositive patients were negative or borderline for neutralizing antibodies to SARS-CoV-2. In this subset of patients, CAS+IMD significantly reduced viral load compared to placebo. In contrast, in seropositive patients with measurable neutralizing activity, no significant impact on viral load was observed in CAS+IMD-treated patients relative to placebo. Furthermore, in the subset of seropositive patients who were negative or borderline for neutralizing antibodies, CAS+IMD treatment led to a trend toward benefit in death or mechanical ventilation as well as mortality compared to placebo.

In the subset of seropositive patients who were positive for neutralizing antibodies, CAS+IMD did not meaningfully impact death or mechanical ventilation or all-cause mortality compared to placebo, but no harm was observed. In contrast, a recent report of bamlanivimab treatment in hospitalized patients with COVID-19 from the ACTIV-3 study raised the question of whether that treatment might have caused harm in the patients who had already mounted an endogenous immune response to SARS-CoV-2 (17). In that analysis, when outcomes were analyzed by functional neutralizing antibody status, both death and the composite safety outcome (death, SAEs, organ failure, and serious coinfections) appeared to be worse in those who were positive for neutralizing antibodies.

While it is difficult to compare the findings from the ACTIV-3 study to the present analysis, given sample size limitations and differences in SARS-CoV-2 neutralization assays, both analyses indicate a clear trend toward benefit of monoclonal antibody therapy in patients lacking neutralizing antibody activity against SARS-CoV-2. The current study highlights that a subset of seropositive patients who lack neutralizing function against SARS-CoV-2 could still benefit from monoclonal antibody therapy.

Although the number of safety events was small, they were consistent with the primary analysis (12), and we did not observe any new or unknown safety signals or clustering in a particular treatment group and/or subset evaluated in this analysis. In the subset of seropositive patients who were negative or borderline for neutralizing antibodies, death rates were lower in the CAS+IMD group compared with the placebo group. This trend was not observed in the subset of seropositive patients who were positive for neutralizing antibodies at baseline. Finally, consistent with previous reports (12), we observed increased AESIs in the CAS+IMD group versus placebo in both subsets of seropositive patients.

As part of this analysis, we further characterized the serological status of seropositive patients, which was defined by a composite of three binding assays to detect antibodies against SARS-CoV-2 by looking at individual assay positivity as well as combinations of assay positivity (see Tables S1 and S2 in the supplemental material). While our data indicated that IgG assays may better differentiate neutralizing negative or borderline patients from neutralizing positive patients versus the composite of assays, there remain subsets of IgG-seropositive patients lacking neutralizing activity who may benefit from treatment with CAS+IMD.

A key observation from this analysis is that utilizing serologic status alone (i.e., seronegativity as measured by an antibody-binding assay) to guide treatment decisions for patients with COVID-19 may fail to identify seropositive patients who may benefit from treatment with monoclonal antibodies, depending on the neutralizing potency of their endogenous antibodies. Moreover, functional antibodies generated from vaccination or natural infection with one variant of SARS-CoV-2 may not be effective against infection from a different variant. This clinical trial was conducted prior to the emergence of Omicron-lineage variants. While CAS+IMD is no longer in use in the United States because of the predominance of Omicron (18), this analysis suggests that the next generation of monoclonal antibodies for the treatment of COVID-19 may benefit patients whose endogenous antibodies lack anti-SARS-CoV-2-neutralizing activity, regardless of the overall baseline serostatus. Given that efficacy of COVID-19 vaccination wanes over time (1, 3, 19), the complexities of virus evolution (including regional differences), and the lack of available rapid point-of-care serology tests that reliably measure neutralizing function, identifying seropositive patients who would potentially benefit from treatment with monoclonal antibodies poses a challenge for patient care.

While we observed trends in improvement in both virological and clinical endpoints in seropositive patients who were negative or borderline for neutralizing antibodies, this is a *post hoc* analysis. Thus, all *P* values are considered nominal. Additionally, there was a relatively small number of patients in the seropositive neutralizing negative or borderline subset (*n *=* *178) compared to those in the neutralizing positive subset, as well as compared to the number of seronegative patients.

Unlike the time of the pandemic during which study 2066 was conducted, the majority of immunocompetent individuals are now expected to be seropositive for antibodies against SARS-CoV-2. The data presented here demonstrate that, in addition to the clinical benefit in seronegative hospitalized patients (11, 12), there may be a clinical benefit of anti-SARS-CoV-2 monoclonal antibody therapies in a subset of seropositive hospitalized patients who lack adequate neutralizing activity to the variant with which they are infected. Thus, regardless of serostatus, further study of the therapeutic potential of monoclonal antibodies with activity against currently circulating SARS-CoV-2 variants is warranted.

## MATERIALS AND METHODS

### Trial design.

The design of this adaptive, phase 1, 2, and 3, double-blind, placebo-controlled trial to evaluate the efficacy, safety, and tolerability of CAS+IMD in hospitalized adult patients with COVID-19 (ClinicalTrials registration no. NCT04426695) has been previously described (12). Briefly, patients were enrolled in one of the following four cohorts based on disease severity: no supplemental oxygen (cohort 1A), low-flow oxygen (cohort 1), high-intensity oxygen (cohort 2), or mechanical ventilation (cohort 3). The trial proceeded through phase 2 for patients requiring no supplemental oxygen and phase 3 for patients requiring low-flow oxygen (O_2_ saturation of >93% on low-flow oxygen via nasal cannula, simple face mask, or other similar device); together, these patients are the subject of the manuscript. For patients requiring high-intensity oxygen or mechanical ventilation, enrollment was paused early in the study per independent data monitoring committee (IDMC) recommendation as previously described (12), and these data are not included in the manuscript.

As previously described (12), patients were randomized 1:1:1 to a single intravenous dose of 2.4 g CAS+IMD, 8.0 g CAS+IMD, or placebo. The trial included a screening/baseline period, a hospitalization/postdischarge period (days 1 to 29), a monthly follow-up period, and an end-of-study visit (phase 1, day 169; phases 2 and 3, day 57).

### Patients.

Patients were ≥18 years of age and hospitalized with confirmed SARS-CoV-2 within 72 h of randomization and with symptom onset ≤10 days from randomization. Standard-of-care treatments for COVID-19 were permitted per the investigator. Full inclusion and exclusion criteria have been previously described (12). SARS-CoV-2 infection and baseline viral load were determined as previously described (Eurofins Viracor BioPharma Services, Inc., Lee’s Summit, MO, USA) (20).

### SARS-CoV-2 serostatus.

All patients were assessed for the presence or absence of anti-SARS-CoV-2 antibodies at baseline by the following three assays, comprising a composite serostatus: anti-spike S1 IgA (Euroimmun), anti-spike S1 IgG (Euroimmun), and anti-nucleocapsid IgG (Abbott). The serology assays at baseline were run at a central laboratory (Icon Central Laboratories, Farmingdale, NY, USA). Patients underwent randomization regardless of their baseline serostatus and were grouped for analyses as seropositive (if any baseline antibody test was positive) or seronegative (if all available baseline antibody tests were negative). Subjects who either had a borderline serostatus (if any test result was borderline in the absence of any positive test result) or their test results were missing, not determined, or pending were categorized as other.

### SARS-CoV-2 neutralization status.

SARS-CoV-2 functional neutralizing titers were determined using a validated recombinant VSV neutralization assay where the VSV glycoprotein (G) was replaced by the SARS-CoV-2 spike (S) protein (utilizing the Wuhan sequence, GenBank accession no. NC_045512) with a luciferase activity readout (Vyriad, Inc. and Imanis Life Sciences, LLC, Rochester, MN, USA) (21). In brief, human serum samples were mixed with virus (VSV-SARS-CoV-2-S-D19CT) and incubated at room temperature for 30 min. Samples were assayed at 1:80, 1:160, 1:320, 1:640, 1:1,280, and 1:2,560 and heat inactivated (56°C, 30 min). Pooled SARS-CoV-2-seronegative sera with or without virus were used as negative nonneutralizing controls. Virus plus SARS-CoV-2-seronegative serum spiked with either 0.4 μg/mL or 0.6 μg/mL of an anti-SARS-CoV-2 spike-neutralizing monoclonal antibody were used as a low-positive control (LPC) or a high-positive control (HPC), respectively. Serum-virus mixtures were overlaid onto Vero-DSP-1/2 monolayers and placed in a 37°C and 5% CO_2_ incubator. Bioluminescence was read 20 to 24 h after virus-serum overlay on the cells. Percent signals for each serum sample were determined from corrected raw light values based on negative controls. For analysis, patients were resulted as neutralizing positive (percent signal below HPC), neutralizing negative (percent signal above low-positive control LPC), neutralizing borderline (percent signal between HPC and LPC), or neutralizing indeterminant (percent signal for one replicate above LPC and one replicate below LPC).

### Outcome measures.

In this *post hoc* analysis, the following efficacy endpoints were evaluated in seropositive patients by neutralizing antibody status: (i) TWA daily change from baseline (day 1) in viral load in nasopharyngeal samples through day 11, (ii) the proportion of patients who died or required mechanical ventilation from baseline (day 1) to day 29, and (iii) the proportion of patients who died (all-cause mortality) from baseline (day 1) to day 29.

The following *post hoc* safety endpoints were also evaluated in seropositive patients by neutralizing antibody status: the proportion of patients with (i) treatment-emergent SAEs through the end of the study, and (ii) AESIs, specifically grade ≥2 infusion-related reactions through day 4 and grade ≥2 hypersensitivity reactions through day 29.

### Statistical analysis.

As previously described (12), enrollment in this study was terminated on 9 April 2021 for strategic reasons and was not based on any safety concerns. Accordingly, enrollment of patients receiving low-flow (cohort 1) and no supplemental oxygen (cohort 1A) was prematurely terminated, but all ongoing patients were followed up through the end of the study. This resulted in smaller than planned sample size. As such, phase 1, 2, and 3 patients on low-flow oxygen (cohort 1) were pooled with phase 2 patients with no supplemental oxygen (cohort 1A) for the current analysis. Additionally, it was elected to combine the CAS+IMD 2.4-g and 8.0-g dose groups for analysis. As previously described in the primary analysis publication (12), sensitivity analyses did not reveal substantial efficacy differences across the cohorts or doses.

The FAS includes all randomized patients who received any amount of study drug. The mFAS includes all FAS patients who had a positive central lab SARS-CoV-2 quantitative reverse transcriptase PCR result at baseline. The seropositive mFAS includes all patients in the mFAS who were grouped for analysis as seropositive as described above; this population was used for all efficacy and safety analyses.

TWA daily change from baseline in viral load was analyzed using the analysis of covariance model, as previously described (12). The proportion of patients who died or required mechanical ventilation, as well as all-cause mortality, was analyzed using either the exact method for binomial distribution or asymptotic normal approximation method as previously described (12). Cumulative incidence of death or mechanical ventilation as well as incidence of death are depicted as Kaplan-Meier curves. Safety endpoints were analyzed descriptively.

All reported *P* values for this *post hoc* analysis are nominal. Missing data were handled as previously described (12).

### Trial oversight.

Regeneron Pharmaceuticals, Inc. designed the trial and, with the trial investigators, gathered the data. Regeneron Pharmaceuticals, Inc. analyzed the data. The list of trial investigators has been previously described (12). The investigators, site personnel, and Regeneron Pharmaceuticals, Inc. were unaware of the treatment group assignments. An IDMC monitored unblinded data to make recommendations about trial modifications.

The trial was conducted in accordance with the principles of the Declaration of Helsinki, the International Council for Harmonisation Good Clinical Practice Guidelines, and applicable regulatory requirements. The local institutional review board or ethics committee at each study center oversaw trial conduct and documentation. All patients provided written informed consent before participating in the trial.

### Data availability.

Qualified researchers may request access to study documents (including the clinical study report, study protocol with any amendments, blank case report form, and statistical analysis plan) that support the methods and findings reported in the manuscript. Individual anonymized participant data will be considered for sharing once the product and indication have been approved by major health authorities (e.g., Food and Drug Administration, European Medicines Agency, Pharmaceuticals and Medical Devices Agency, etc.) if there is legal authority to share the data and there is not a reasonable likelihood of participant reidentification. Requests should be submitted to https://vivli.org/.
