# Supplementary material for: Casirivimab and Imdevimab Treatment Reduces Viral Load and Improves Clinical Outcomes in Seropositive Hospitalized COVID-19 Patients with Nonneutralizing or Borderline Neutralizing Antibodies
Source: mBio. 2022 Oct 18;13(6):e01699-22. doi: 10.1128/mbio.01699-22 (PMC9765482; doi:10.1128/mbio.01699-22)
Supplement: FIG S1 [file mbio.01699-22-s0001.pdf]

FIG S1 Flow diagram for the phase 1/2/3 population receiving low-flow or no supplemental oxygen

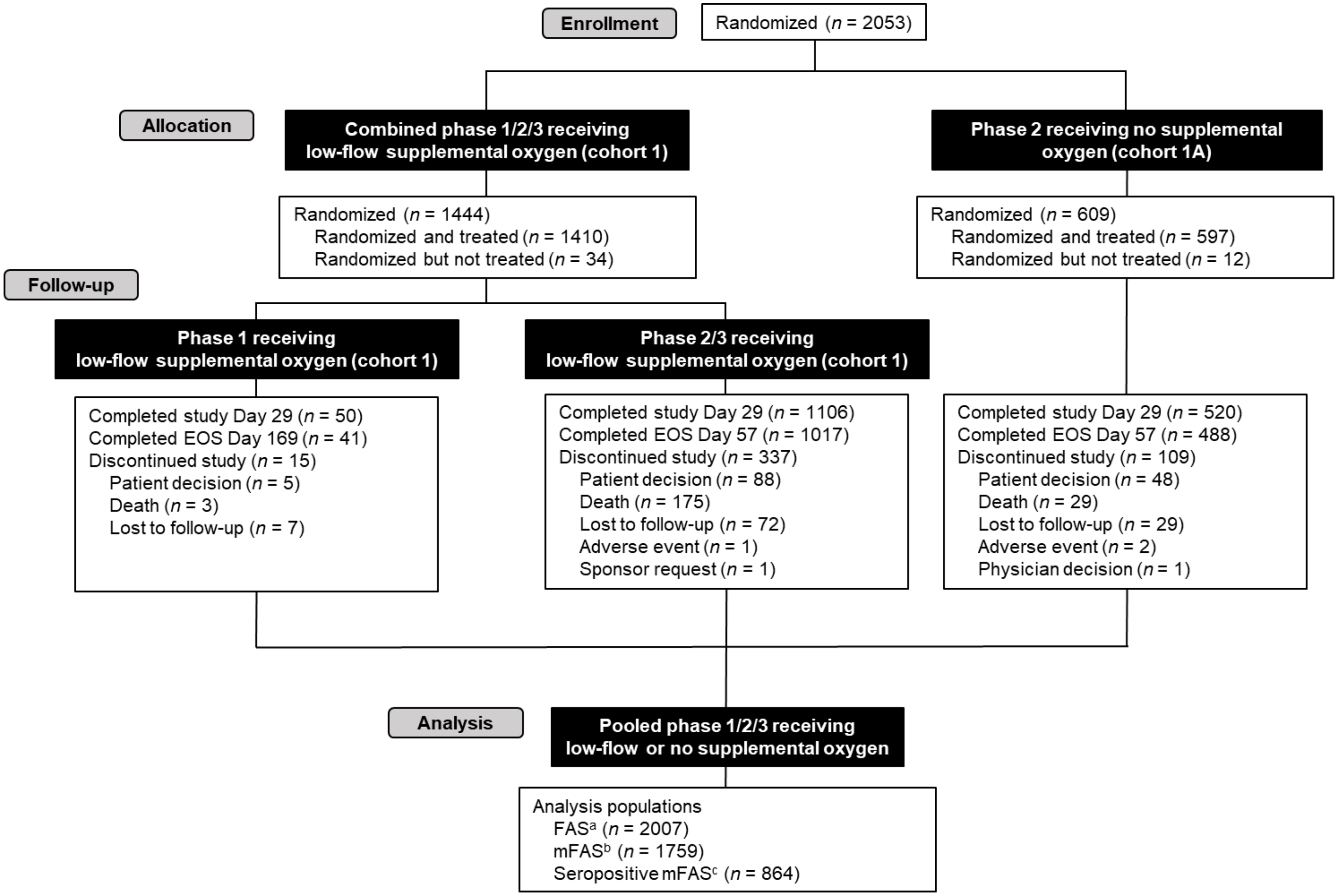

[a]The FAS includes all randomized patients who received at least one dose (full or partial) of the study drug. Analysis of the FAS population was according to the treatment allocated (as randomized).

[b]The mFAS includes all FAS patients with a positive SARS-CoV-2 RT-qPCR conducted in the central laboratory in nasopharyngeal swab samples at randomization, and analysis is based on the treatment allocated (as randomized).

[c]The seropositive mFAS is defined as all patients in mFAS in whom any baseline serology test (anti-spike [S1] IgA, anti-spike [S1] IgG, or anti-nucleocapsid IgG) was positive.

EOS, end of study; FAS, full analysis set; Ig, immunoglobulin; mFAS, modified full analysis set; RT-qPCR, quantitative reverse transcription polymerase chain reaction; SARS-CoV-2, severe acute respiratory syndrome coronavirus 2.
