# Supplementary material for: Casirivimab and Imdevimab Treatment Reduces Viral Load and Improves Clinical Outcomes in Seropositive Hospitalized COVID-19 Patients with Nonneutralizing or Borderline Neutralizing Antibodies
Source: mBio. 2022 Oct 18;13(6):e01699-22. doi: 10.1128/mbio.01699-22 (PMC9765482; doi:10.1128/mbio.01699-22)
Supplement: TABLE S1 [file mbio.01699-22-s0002.pdf]

TABLE S1 Characterization of neutralization status in seropositive patients by individual serology assays[a]

Seroassay	patients, *n*	Seropositive patients positive for each assay, *n/N* (%)	Neutralization status, *n1/n* (%)				
			Positive	Negative	Borderline	Negative or borderline	U/M/I
IgA spike	764	764/1705[b] (44.8)	587/764 (76.8)	107/764 (14.0)	31/764 (4.1)	138/764 (18.1)	39/764 (5.1)
IgG spike	372	372/1705[b] (21.8)	328/372 (88.2)	23/372 (6.2)	4/372 (1.1)	27/372 (7.3)	17/372 (4.6)
IgG NC	534	534/1710[b] (31.2)	446/534 (83.5)	44/534 (8.2)	21/534 (3.9)	65/534 (12.2)	23/534 (4.3)

[a]Seropositive mFAS presented.

[b]Denominators differ based on the number of patients with available data for each assay.

Ig, immunoglobulin; mFAS, modified full analysis set; NC, nucleocapsid; U/M/I, unknown/missing/indeterminate.
