# Supplementary material for: Casirivimab and Imdevimab Treatment Reduces Viral Load and Improves Clinical Outcomes in Seropositive Hospitalized COVID-19 Patients with Nonneutralizing or Borderline Neutralizing Antibodies
Source: mBio. 2022 Oct 18;13(6):e01699-22. doi: 10.1128/mbio.01699-22 (PMC9765482; doi:10.1128/mbio.01699-22)
Supplement: TABLE S2 [file mbio.01699-22-s0003.pdf]

TABLE S2 Characterization of subgroup seropositivity by neutralization status[a]

Neutralizing status	Patients, *n*	Patients positive for each assay combination as presented, *n/N* (%)							
		IgA spike[b]	IgG spike[b]	IgG NC[b]	IgA spike and IgG spike[c]	IgA spike and IgG NC[c]	IgG spike and IgG NC[c]	IgG spike and/or IgG NC[d]	Triple positive[e]
Negative or borderline	178	91/178 (51.1)	11/178 (6.2)	27/178 (15.2)	11/178 (6.2)	33/178 (18.5)	2/178 (1.1)	40/178 (22.4)	3/178 (1.7)
Negative	138	75/138 (54.3)	10/138 (7.2)	19/138 (13.8)	9/138 (6.5)	21/138 (15.8)	2/138 (1.4)	31/138 (22.4)	2/138 (1.4)
Borderline	40	16/40 (40.0)	1/40 (2.5)	8/40 (20.0)	2/40 (5.0)	12/40 (30.0)	0 (0)	9/40 (22.5)	1/40 (2.5)
Positive	643	145/643 (22.6)	8/643 (1.2)	39/643 (6.1)	44/643 (6.8)	131/643 (20.4)	9/643 (1.4)	56/643 (8.6)	267/643 (41.5)

[a]Seropositive mFAS presented.

[b]Positive for only the individual assay specified.

[c]Positive for both of the assays specified but not positive for the remaining assay.

[d]Positive for either or both of the IgG assays (IgG spike, IgG NC) but not positive for IgA spike.

[e]Positive for all of the following three assays: IgA spike, IgG spike, and IgG NC.

Ig, immunoglobulin; mFAS, modified full analysis set; NC, nucleocapsid.
