# Supplementary material for: Casirivimab and Imdevimab Treatment Reduces Viral Load and Improves Clinical Outcomes in Seropositive Hospitalized COVID-19 Patients with Nonneutralizing or Borderline Neutralizing Antibodies
Source: mBio. 2022 Oct 18;13(6):e01699-22. doi: 10.1128/mbio.01699-22 (PMC9765482; doi:10.1128/mbio.01699-22)
Supplement: TABLE S3 [file mbio.01699-22-s0004.pdf]

TABLE S3 Time-weighted average change in viral load from baseline in seropositive patients by baseline neutralizing antibody status[a,b]

	**Negative or borderline for neutralizing antibodies**				**Positive for neutralizing antibodies**			
	**Placebo (*n* = 68)**	**CAS+IMD 2.4 g IV (*n* = 57)**	**CAS+IMD 8.0 g IV (*n* = 53)**	**CAS+IMD combined (*n* = 110)**	**Placebo (*n* = 222)**	**CAS+IMD 2.4 g IV (*n* = 213)**	**CAS+IMD 8.0 g IV (*n* = 208)**	**CAS+IMD combined (*n* = 421)**
Baseline (Day 1) to Day 3								
Patients, *n*	57	41	43	84	181	167	171	338
LS mean change (SE), log_{10} copies/mL	–0.27 (0.11)	–0.64 (0.13)	–0.67 (0.12)	–0.66 (0.09)	–0.43 (0.06)	–0.52 (0.06)	–0.46 (0.06)	–0.49 (0.04)
95% CI	–0.48, –0.05	–0.89, –0.39	–0.92, –0.42	–0.83, –0.48	–0.55, –0.31	–0.65, –0.40	–0.58, –0.33	–0.58, –0.40
Difference versus placebo, log_{10} copies/mL								
LS mean (SE)	–	–0.37 (0.17)	–0.40 (0.16)	–0.39 (0.14)	–	–0.09 (0.09)	–0.03 (0.09)	–0.06 (0.08)
95% CI	–	–0.70, –0.04	–0.73, –0.07	–0.66, –0.11	–	–0.27, 0.08	–0.20, 0.14	–0.21, 0.09
Nominal *P* value	–	**0.0270**	**0.0163**	**0.0061**	–	0.2894	0.7425	0.4248
Baseline (Day 1) to Day 5								
Patients, *n*	61	44	47	91	195	186	182	368

LS mean change (SE), log_{10} copies/mL	–0.52 (0.13)	–1.02 (0.15)	–1.12 (0.14)	–1.07 (0.10)	–0.72 (0.07)	–0.92 (0.07)	–0.73 (0.07)	–0.83 (0.05)
95% CI	–0.77, –0.27	–1.32, –0.73	–1.40, –0.83	–1.27, –0.87	–0.86, –0.59	–1.06, –0.78	–0.87, –0.59	–0.93, –0.73
Difference versus placebo, log_{10} copies/mL	–							
LS mean (SE)	–	–0.50 (0.20)	–0.60 (0.19)	–0.55 (0.16)	–	–0.20 (0.10)	–0.01 (0.10)	–0.10 (0.09)
95% CI	–	–0.89, –0.11	–0.97, –0.22	–0.87, –0.23	–	–0.39, 0.00	–0.21, 0.19	–0.27, 0.07
Nominal *P* value	–	**0.0113**	**0.0021**	**0.0009**	–	0.0530	0.9416	0.2412
Baseline (Day 1) to Day 7								
Patients, *n*	61	49	50	99	201	193	184	377
LS mean change (SE), log_{10} copies/mL	–0.81 (0.13)	–1.32 (0.15)	–1.49 (0.15)	–1.40 (0.10)	–0.93 (0.07)	–1.23 (0.07)	–0.99 (0.07)	–1.11 (0.05)
95% CI	–1.07, –0.55	–1.61, –1.02	–1.78, –1.20	–1.61, –1.19	–1.07, –0.79	–1.37, –1.09	–1.14, –0.85	–1.22, –1.01
Difference versus placebo, log_{10} copies/mL								
LS mean (SE)	–	–0.51 (0.20)	–0.68 (0.20)	–0.59 (0.17)	–	–0.30 (0.10)	–0.06 (0.10)	–0.18 (0.09)
95% CI	–	–0.90, –0.11	–1.07, –0.29	–0.92, –0.26	–	–0.50, –0.09	–0.26, 0.14	–0.36, –0.01
Nominal *P* value	–	**0.0123**	**0.0008**	**0.0006**	–	**0.0041**	**0.5592**	**0.0431**

Baseline (Day 1) to Day 9								
Patients, *n*	64	51	50	101	202	193	187	380
LS mean change (SE), $\log_{10}$ copies/mL	–1.11 (0.15)	–1.63 (0.16)	–1.76 (0.16)	–1.69 (0.12)	–1.18 (0.08)	–1.48 (0.08)	–1.23 (0.08)	–1.35 (0.06)
95% CI	–1.40, –0.82	–1.95, –1.31	–2.09, –1.44	–1.92, –1.47	–1.33, –1.03	–1.63, –1.32	–1.39, –1.08	–1.46, –1.25
Difference versus placebo, $\log_{10}$ copies/mL								
LS mean (SE)	–	–0.52 (0.22)	–0.65 (0.22)	–0.58 (0.19)	–	–0.30 (0.11)	–0.05 (0.11)	–0.18 (0.09)
95% CI	–	–0.95, –0.09	–1.08, –0.22	–0.95, –0.22	–	–0.51, –0.09	–0.27, 0.16	–0.36, 0.01
Nominal *P* value	–	**0.0186**	**0.0035**	**0.0020**	–	0.0059	0.6180	0.0580
Baseline (Day 1) to Day 11								
Patients, *n*	64	51	50	101	203	194	189	383
LS mean change (SE), $\log_{10}$ copies/mL	–1.33 (0.15)	–1.83 (0.17)	–1.92 (0.17)	–1.87 (0.12)	–1.36 (0.08)	–1.66 (0.08)	–1.41 (0.08)	–1.54 (0.06)
95% CI	–1.64, –1.03	–2.17, –1.49	–2.27, –1.58	–2.11, –1.63	–1.52, –1.20	–1.82, –1.50	–1.57, –1.25	–1.65, –1.42
Difference versus placebo, $\log_{10}$ copies/mL								
LS mean (SE)	–	–0.49 (0.23)	–0.59 (0.23)	–0.54 (0.20)	–	–0.30 (0.11)	–0.05 (0.12)	–0.18 (0.10)

95% CI	–	–0.95, –0.04	–1.05, –0.13	–0.93, –0.15	–	–0.52, –0.07	–0.28, 0.18	–0.37, 0.02
Nominal *P* value	–	**0.0346**	**0.0124**	**0.0067**	–	**0.0092**	0.6605	0.0765

[a]Seropositive mFAS presented.

[b]Nominal *P* values >0.05 are in bold.

CAS+IMD, casirivimab and imdevimab; CI, confidence interval; IV, intravenous; LS, least squares; mFAS, modified full analysis set.
