# Supplementary material for: Casirivimab and Imdevimab Treatment Reduces Viral Load and Improves Clinical Outcomes in Seropositive Hospitalized COVID-19 Patients with Nonneutralizing or Borderline Neutralizing Antibodies
Source: mBio. 2022 Oct 18;13(6):e01699-22. doi: 10.1128/mbio.01699-22 (PMC9765482; doi:10.1128/mbio.01699-22)
Supplement: TABLE S4 [file mbio.01699-22-s0005.pdf]

TABLE S4 Clinical outcomes in seropositive patients by baseline neutralizing antibody status[a]

	**Negative or borderline for neutralizing antibodies**				**Positive for neutralizing antibodies**			
	**Placebo (*n* = 68)**	**CAS+IMD 2.4 g IV (*n* = 57)**	**CAS+IMD 8.0 g IV (*n* = 53)**	**CAS+IMD combined (*n* = 110)**	**Placebo (*n* = 222)**	**CAS+IMD 2.4 g IV (*n*= 213)**	**CAS+IMD 8.0 g IV (*n* = 208)**	**CAS+IMD combined (*n*= 421)**
Proportion of patients who died or went on mechanical ventilation from baseline (Day 1) to Day 29								
n/total *N* (%)	13/68 (19.1)	10/57 (17.5)	2/53 (3.8)	12/110 (10.9)	18/222 (8.1)	13/213 (6.1)	21/208 (10.1)	34/421 (8.1)
Relative risk reduction vs placebo, %	–	8.2	80.3	42.9	–	24.7	–24.5	0.4
95% CI, %	–	–93.4, 56.5	16.3, 95.3	–17.7, 72.3	–	–49.8, 62.2	–127.0, 31.7	–72.2, 42.4
Nominal *P* value	–	0.7568	0.0122	0.1125	–	0.3537	0.5455	0.8689
Proportion of patients who died from baseline (Day 1) to Day 29								
n/total *N* (%)	11/68 (16.2)	8/57 (14.0)	2/53 (3.8)	10/110 (9.1)	12/222 (5.4)	8/213 (3.8)	17/208 (8.2)	25/421 (5.9)
Relative risk reduction vs placebo, %	–	13.2	76.7	43.8	–	30.5	–51.2	–9.9
95% CI, %	–	–101.0, 62.5	–0.8, 94.6	–25.2, 74.8	–	–66.6, 71.0	–208.9, 26.0	–114.4, 43.7
Nominal *P* value	–	0.5985	0.0377	0.1190	–	0.3409	0.2964	0.8893

[a]Seropositive mFAS presented.

CAS+IMD, casirivimab and imdevimab; CI, confidence interval; IV, intravenous; mFAS, modified full analysis set.
