# Supplementary material for: Casirivimab and Imdevimab Treatment Reduces Viral Load and Improves Clinical Outcomes in Seropositive Hospitalized COVID-19 Patients with Nonneutralizing or Borderline Neutralizing Antibodies
Source: mBio. 2022 Oct 18;13(6):e01699-22. doi: 10.1128/mbio.01699-22 (PMC9765482; doi:10.1128/mbio.01699-22)
Supplement: TABLE S5 [file mbio.01699-22-s0006.pdf]

TABLE S5 Adverse events leading to death in seropositive patients by baseline neutralizing antibody status[a]

**Primary system organ class**<br>**Preferred term**	**Placebo**	**CAS+IMD 2.4 g IV**	**CAS+IMD 8.0 g IV**	**CAS+IMD combined doses**
**Baseline neutralizing antibody status: Negative or borderline**	***n* = 68**	***n* = 57**	***n* = 53**	***n* = 110**
TEAEs leading to death, n	12	9	2	11
Patients with at least one TEAE leading to death, n (%)	12 (17.6)	9 (15.8)	2 (3.8)	11 (10.0)
Cardiac disorders, *n* (%)	1 (1.5)	3 (5.3)	0	3 (2.7)
Cardiac arrest	1 (1.5)	1 (1.8)	0	1 (0.9)
Cardiogenic shock	0	1 (1.8)	0	1 (0.9)
Ventricular tachycardia	0	1 (1.8)	0	1 (0.9)
Infections and infestations, *n* (%)	6 (8.8)	1 (1.8)	2 (3.8)	3 (2.7)
COVID-19	1 (1.5)	1 (1.8)	1 (1.9)	2 (1.8)
COVID-19 pneumonia	3 (4.4)	0	1 (1.9)	1 (0.9)
Pulmonary sepsis	2 (2.9)	0	0	0
Respiratory, thoracic, and mediastinal disorders, n (%)	3 (4.4)	3 (5.3)	0	3 (2.7)
Acute respiratory failure	1 (1.5)	3 (5.3)	0	3 (2.7)
Idiopathic pulmonary fibrosis	1 (1.5)	0	0	0
Respiratory failure	1 (1.5)	0	0	0

General disorders and administration site conditions, *n* (%)	2 (2.9)	2 (3.5)	0	2 (1.8)
Death	1 (1.5)	2 (3.5)	0	2 (1.8)
Multiple organ dysfunction syndrome	1 (1.5)	0	0	0
**Baseline neutralizing antibody status: Positive**	***n* = 222**	***n* = 213**	***n* =*n*208**	***n* = 421**
TEAEs leading to death, n	14	11	20	31
Patients with at least one TEAE leading to death, n (%)	14 (6.3)	11 (5.2)	19 (9.1)	30 (7.1)
Respiratory, thoracic, and mediastinal disorders, *n* (%)	4 (1.8)	4 (1.9)	6 (2.9)	10 (2.4)
Acute respiratory failure	0	1 (0.5)	3 (1.4)	4 (1.0)
Respiratory failure	2 (0.9)	1 (0.5)	3 (1.4)	4 (1.0)
Pulmonary artery thrombosis	0	1 (0.5)	0	1 (0.2)
Pulmonary embolism	0	1 (0.5)	0	1 (0.2)
Hypoxia	1 (0.5)	0	0	0
Pneumonitis	1 (0.5)	0	0	0
General disorders and administration site conditions, *n* (%)	1 (0.5)	2 (0.9)	6 (2.9)	8 (1.9)
Multiple organ dysfunction syndrome	1 (0.5)	2 (0.9)	6 (2.9)	8 (1.9)
Infections and infestations, *n* (%)	5 (2.3)	2 (0.9)	5 (2.4)	7 (1.7)
COVID-19	3 (1.4)	0	3 (1.4)	3 (0.7)
COVID-19 pneumonia	0	0	1 (0.5)	1 (0.2)
Pneumonia	0	0	1 (0.5)	1 (0.2)

Septic shock	1 (0.5)	1 (0.5)	0	1 (0.2)
Urosepsis	0	1 (0.5)	0	1 (0.2)
Pulmonary sepsis	1 (0.5)	0	0	0
Cardiac disorders, *n* (%)	3 (1.4)	2 (0.9)	3 (1.4)	5 (1.2)
Cardio-respiratory arrest	1 (0.5)	1 (0.5)	2 (1.0)	3 (0.7)
Acute myocardial infarction	0	0	1 (0.5)	1 (0.2)
Cardiac arrest	0	1 (0.5)	0	1 (0.2)
Acute left ventricular failure	1 (0.5)	0	0	0
Atrial fibrillation	1 (0.5)	0	0	0
Nervous system disorders, *n* (%)	1 (0.5)	1 (0.5)	0	1 (0.2)
Metabolic encephalopathy	0	1 (0.5)	0	1 (0.2)
Dementia Alzheimer's type	1 (0.5)	0	0	0

[a]Seropositive mFAS presented.

A patient who reported two or more adverse events with different preferred terms within the same system organ class is counted only once in that system organ class. A patient who reported two or more adverse events with the same preferred term is counted only once for that term.

Primary system organ classes are sorted according to decreasing order of frequency of all treatment groups combined. Within each system organ class, preferred terms are sorted by decreasing frequency.

CAS+IMD, casirivimab and imdevimab; COVID-19, coronavirus disease 2019; IV, intravenous; mFAS, modified full analysis set.
