# Supplementary material for: Casirivimab and Imdevimab Treatment Reduces Viral Load and Improves Clinical Outcomes in Seropositive Hospitalized COVID-19 Patients with Nonneutralizing or Borderline Neutralizing Antibodies
Source: mBio. 2022 Oct 18;13(6):e01699-22. doi: 10.1128/mbio.01699-22 (PMC9765482; doi:10.1128/mbio.01699-22)
Supplement: TABLE S6 [file mbio.01699-22-s0007.pdf]

TABLE S6 Adverse events of special interest in seropositive patients by baseline neutralizing antibody status[a]

**Primary system organ class**<br>**Preferred term**	**Placebo**	**CAS+IMD 2.4 g IV**	**CAS+IMD 8.0 g IV**	**CAS+IMD combined doses**
**Baseline neutralizing antibody status: Negative or borderline**	***n* = 68**	***n* = 57**	***n* = 53**	***n*= 110**
Number of AESIs	0	2	9	11
Number of patients with at least one AESI (%)	0	2 (3.5)	5 (9.4)	7 (6.4)
General disorders and administration site conditions, *n* (%)	0	1 (1.8)	3 (5.7)	4 (3.6)
Chills	0	0	2 (3.8)	2 (1.8)
Edema	0	0	1 (1.9)	1 (0.9)
Systemic inflammatory response syndrome	0	1 (1.8)	0	1 (0.9)
Respiratory, thoracic, and mediastinal disorders, *n* (%)	0	1 (1.8)	1 (1.9)	2 (1.8)
Hypoxia	0	1 (1.8)	1 (1.9)	2 (1.8)
Cardiac disorders, *n* (%)	0	0	1 (1.9)	1 (0.9)
Tachycardia	0	0	1 (1.9)	1 (0.9)
Gastrointestinal disorders, *n* (%)	0	0	1 (1.9)	1 (0.9)
Nausea	0	0	1 (1.9)	1 (0.9)
Vomiting	0	0	1 (1.9)	1 (0.9)
Immune system disorders, *n* (%)	0	0	1 (1.9)	1 (0.9)

Anaphylactic reaction	0	0	1 (1.9)	1 (0.9)
Nervous system disorders, *n* (%)	0	0	1 (1.9)	1 (0.9)
Headache	0	0	1 (1.9)	1 (0.9)
**Baseline neutralizing antibody status: Positive**	***n* = 222**	***n* = 213**	***n* =208**	***n* = 421**
Number of AESIs	1	5	3	8
Number of patients with at least one AESI (%)	1 (0.5)	4 (1.9)	3 (1.4)	7 (1.7)
Nervous system disorders, *n* (%)	0	1 (0.5)	1 (0.5)	2 (0.5)
Dizziness	0	0	1 (0.5)	1 (0.2)
Hypoesthesia	0	1 (0.5)	0	1 (0.2)
Paresthesia	0	1 (0.5)	0	1 (0.2)
Respiratory, thoracic, and mediastinal disorders, *n* (%)	0	1 (0.5)	1 (0.5)	2 (0.5)
Dyspnea	0	0	1 (0.5)	1 (0.2)
Hypoxia	0	1 (0.5)	0	1 (0.2)
General disorders and administration site conditions, *n* (%)	0	0	1 (0.5)	1 (0.2)
Infusion site pain	0	0	1 (0.5)	1 (0.2)
Investigations, *n* (%)	0	1 (0.5)	0	1 (0.2)
Aspartate aminotransferase increased	0	1 (0.5)	0	1 (0.2)
Skin and subcutaneous tissue disorders, *n* (%)	0	1 (0.5)	0	1 (0.2)
Pruritus	0	1 (0.5)	0	1 (0.2)

Injury, poisoning, and procedural complications, *n* (%)	1 (0.5)	0	0	0
Infusion-related reaction	1 (0.5)	0	0	0

[a]Seropositive mFAS presented.

A patient who reported two or more adverse events with different preferred terms within the same system organ class is counted only once in that system organ class. A patient who reported two or more adverse events with the same preferred term is counted only once for that term.

Primary system organ classes are sorted according to decreasing order of frequency of all treatment groups combined. Within each system organ class, preferred terms are sorted by decreasing frequency.

AESI, adverse event of special interest; CAS+IMD, casirivimab and imdevimab; IV, intravenous; mFAS, modified full analysis set.
